# Alterations in the Gut Microbiota of Zebrafish (*Danio rerio*) in Response to Water-Soluble Crude Oil Components and Its Mixture With a Chemical Dispersant

**DOI:** 10.3389/fpubh.2020.584953

**Published:** 2020-10-26

**Authors:** Carlos Eduardo González-Penagos, Jesús Alejandro Zamora-Briseño, Daniel Cerqueda-García, Monica Améndola-Pimenta, Juan Antonio Pérez-Vega, Emanuel Hernández-Nuñez, Rossanna Rodríguez-Canul

**Affiliations:** ^1^Departamento de Recursos del Mar, Centro de Investigación y de Estudios Avanzados del Instituto Politécnico Nacional-Unidad Mérida, Mérida, Mexico; ^2^CONACYT – Centro de Investigación y de Estudios Avanzados del Instituto Politécnico Nacional, Mérida, Mexico

**Keywords:** zebrafish, gut microbiota, crude oil, WAF, CEWAF, dysbiosis

## Abstract

Crude oil spills have caused substantial impacts to aquatic ecosystems. Chemical dispersants are used to palliate the impact of oil spillages, but their use is polemic due to their additional potential toxic effect when mixed with oil-derived components. In this work, we used a 16S-based metagenomic approach to analyze the changes of the gut microbiota of adult zebrafish (*Danio rerio*) exposed to the water accommodated fraction (WAF) of a light crude oil (35° API gravity), and the chemically enhanced WAF (CEWAF), prepared with Nokomis 3-F4® dispersant. After 96 h of exposure, WAF induced an increase in the alpha and beta diversity, altering the relative abundance of *Vibrio, Flavobacterium*, and *Novosphingobium*. In contrast, CEWAF only caused an increase in the beta diversity, and an enrichment of the genus *Pseudomona*. Both treatments diminished the abundances of *Aeromonas, Cetobacterium, Coxiella, Dinghuibacter*, and *Paucibacter*. Moreover, the co-occurrence network among genera was more complex in WAF than in CEWAF, indicating a greater bacterial interaction in response to WAF. Our results indicate that short-term exposure to WAF and CEWAF can induce a dysbiosis in the gut microbiota of *D. rerio*, but these changes are specific in each treatment.

## Introduction

Crude oil extraction activities in the Gulf of Mexico (GoM) are source of pollutants (1–3). Crude oil is a complex mixture of low and high molecular weight hydrocarbons, which represent up to 75% of its total composition and includes aliphatic and polycyclic aromatic hydrocarbons (PAHs) (4). In the non-hydrocarbon fraction, crude oil also contains nitrogen, sulfur, oxygen-derived compounds, as well as trace concentrations of heavy metals, such as nickel and iron (5).

Additional to oil pollutants associated to the natural release of petroleum (Spanish = chapopoteras), and those related to anthropogenic activities, hydrocarbons can enter in marine ecosystems during oil spills, as that occurred during the blowout of the Macondo well-operated by the Deepwater Horizon platform (DWH) in 2010. In that event, ~4.4 × 10^6^ oil barrels were released into the sea for 84 days, producing a wide negative impact in the GoM at different biological levels (3).

During oil spill events, chemical dispersants (composed predominantly by surfactants and/or chemical solvents), are frequently used to reduce the tension in the surface of spilled oil by emulsifying their surface and increasing the oil-water solubility (6). For example, during the DWH disaster, ~1.5 M gallons of the chemical dispersant Corexit EC9500® were applied (6). However, the toxicity of the resulting emulsified hydrocarbons involves ecological alterations (7), and constitute a major threat to the environment, especially when they reach coastal areas (5). Another commonly used dispersant is Nokomis 3-F4® (Mar-Len Supply, Inc., Hayward, CA), which is one of the several commercially available formulations recommended for oil spill events, by the United States Environmental Protection Agency (US EPA) (8). The public available composition of this chemical dispersant is very limited, because its formulation is subjected to industrial secret protection (https://www.epa.gov/emergency-response/nokomis-3-F4), but its use is recommended in oil spills produced in fresh water or marine environments. However, some reports have addressed that Nokomis 3-F4® exposure interacts with the estrogen receptor (ER) and the androgen receptor (AR) of some marine organisms (6). This is because, Nokomis 3-F4® contains nonylphenol ethoxylate (NPE) that is degraded to 4-nonylphenol (NP), known as xenoestrogen or endocrine disruptor. This is a compound capable of acting on the endocrine system and altering the reproductive cycle (6). Also, NP can trigger oxidative stress, and it has been associated with obesity-related disorders in several animal models (9, 10).

Crude oil-derived compounds have been associated to carcinogenic, mutagenic, and teratogenic alterations in native aquatic biota (4, 5). Moreover, the exposure to these compounds can negatively impact the symbiotic interactions between host and its associated microorganisms, such as those occupying the gut space.

The gut microbiota maintains a symbiotic relationship with the host (11–13), participating in relevant functions including host metabolism, and immunity (14–16). Microbiota also regulates the function of the intestinal barrier because by having a highly specific composition (16). However, this relationship can be altered either by intrinsic factors of the host and/or by the surrounding environment (17, 18). The imbalance in the gut microbiota, lead to alteration of the host's homeostasis producing a phenomenon known as dysbiosis (17). Changes in the gut microbiota have been used as indicator of chemicals exposure (19–21), since the toxicity of xenobiotics can be modulated after bacterial metabolization (22, 23). In fish, the gut microbiota has been used to assess the effect to exposure of several pollutants, included crude oil exposure (24, 25), antibiotics (26), or PAHs (27), among others. With respect to the study of crude oil, there is still an incomplete comprehension of the effect of crude oil in interaction with chemical dispersants on the gut microbiota of fish. Thereby, we considered that the use of model fish species could help to elucidate in fine detail changes occurring in the gut microbiota in response to crude oil-derived components.

Zebrafish *Danio rerio* is a good candidate in toxicological studies because it possess several advantages; it can be easily manipulated because of its small size, its short generational time, its large number of offspring per laying, as well as the transparency of eggs and embryos (28, 29). It has been used as a model organism for the discovery of pharmacological targets, as well as toxicological evaluation of heavy metals, pesticides, fungicides, nanomaterials, and many other substances. In addition, the ecological dynamics of its gut microbiota communities is well-known (11, 30, 31).

For these reasons, the aim of this study was to evaluate the acute exposure to the water-accommodated fraction (WAF) of a light crude oil, and to the chemically enhanced WAF (CEWAF) with Nokomis 3-F4® on the composition of the gut microbiota of zebrafish.

## Materials and Methods

### Biological Material

One-year-old zebrafish (*D. rerio*) were obtained from the aquaculture facilities of the Center for Research and Advanced Studies of the National Polytechnic Institute-Merida Unit (CINVESTAV-IPN). Fish were kept in UV-treated freshwater in 1 L glass containers with dechlorinated tap water at 27.5 ± 0.5°C and continuous aeration. Fish were fed twice per day *ad libitum* with a commercial fish diet (Wardley®) and maintained at a photoperiod of 14:10 h of light: dark. Previously, this study was reviewed and approved by the Institutional Animal Care and Use Committee of the Center for Research and Advanced Studies (CICUAL-CINVESTAV, approval number: 2875). It complies with the Mexican Official Norm (NOM-062-ZOO-1999), “Technical Specifications for the Care and Use of Laboratory Animals,” as well as all applicable federal and institutional regulations.

### WAF and CEWAF Preparation for Exposure Assays

Light crude oil (extracted from Campo Pool oil-well with 35°API gravity) and Nokomis 3-F4® dispersant were provided by PEMEX Exploration and Production Company. For the exposure assays, we followed the ECETOC (European Center for Ecotoxicology and Toxicology of Chemical Compounds) and the CROSERF (Chemical Response to Oil Spills -Ecological Effects Research Forum) recommendations for the use of water-accommodated fraction (WAF) and chemically enhanced WAF (CEWAF). We followed the method described by Singer et al. (32), with the adaptations proposed by Barron and Ka'aihue (33). WAF was defined as a medium that contains only a soluble fraction of oil that remains in aqueous phase (34). Also, when a dispersant is added to the crude oil-water mixture, a chemically enhanced WAF or CEWAF is obtained. In both cases, only the aqueous phase is used (34).

The WAF and CEWAF mixtures were prepared at the same time. For WAF preparation, crude oil (1 g/L) was added to the filtered, UV-light purified water and placed in a glass jar. For CEWAF preparation, crude oil (1 g/L) was added to the filtered, UV-light purified water and placed in a glass jar. Nokomis 3-F4® was immediately added in a proportion of 1:10 (v/v, dispersant:crude oil).

Both mixtures were prepared in darkness conditions, mixed with a magnetic stirrer for 24 h at 5,000 x rpm to form a vortex equivalent to the 20–25% of the water column height. After this time, the mixtures were let to settle for 1 h, and the lower phase (aqueous phase) for each was collected. These phases were defined as the WAF and CEWAF stock solutions. Then, a 50% dilution (v/v) was prepared from both stock solutions (equal volumes of filtered, UV-light treated water and stock WAF/CEWAF). These dilutions were used for the WAF and CEWAF exposure bioassay, respectively. This sub-lethal concentration of 50% was chosen based on previous data (24).

### WAF/CEWAF Exposure Assays

A 96 h acute static bioassay was performed using 12 male adult zebrafish. Three groups of four zebrafish with similar length and weight were placed individually in 1 L glass aquaria (WAF = 4, CEWAF = 4, and CONTROL = 4). During the bioassay, the zebrafish were kept unfed. Water quality parameters (temperature, dissolved oxygen, salinity, and pH) were measured using an YSI™ 556MPS Multi Probe System multiparameter device (Xylem Inc). In addition, nitrite and ammonium were quantified with an aquaria water quality kit (Nutrafin®). At the end of the exposure time, biometric measures were taken, and the organisms were euthanized by ice water bath immersion for five min. Each fish was surface sanitized with 70% ethanol, and intestines were dissected under aseptic conditions, fixed in five volumes of absolute ethanol, and stored at −80°C until analysis.

### DNA Extraction and 16S rRNA Gene Amplification

Total genomic DNA (gDNA) extraction from the entire intestine of each fish (*n* = 4 per treatment) was performed using the commercial Quick-DNA™ Universal Kit (ZymoResearch^©^). gDNA concentration was determined with a Thermo Scientific NanoDrop™ 2000c spectrophotometer (ThermoFisher Scientific^©^) and integrity checked by 1% agarose gel electrophoresis.

The V3-V4 region of the 16S rRNA bacterial gene was amplified by PCR from the gDNA of each sample. For PCR amplification, we used the 16S rRNA Forward primer: 5' TCGTCGGCAGCGTCAGATGTGTATAAGAGACAGCCTACGGGNGGCWGCAG and 16S rRNA Reverse primer: 5' GTCTCGTGGGCTCGGAGATGTGTATAAGAGACAGGACTACHVGGGTATCTAATCC, which amplify a region of ~550 bp (35). PCRs were performed in a volume of 25 μL containing 12.5 μl of Dream Taq Green PCR Master Mix (2X) (Thermo Scientific^©^), 0.5 μL of gDNA (equivalent to 328 ± 23 ng/μl of gDNA), and 0.115 μL of each primer (125 nM). All reactions were performed in a Thermal cycler C1000 Touch™ (Bio-Rad Laboratories^©^) using a cycling program as follows: initial denaturation of 3 min at 95°C followed by 36 cycles of denaturation for 30 s at 95°C, annealing of 30 s at 53°C, an extension of 60 s at 72°C, and a final extension of 5 min at 72°C. PCR for no-template control was included to guarantee that no cross-tube reactions occurred.

### Library Preparation and Sequencing

Library preparation and sequencing were performed in the National Sequencing and Polymorphisms Detection Unit from the National Institute of Genomic Medicine (INMEGEN), Mexico.

Amplicons clean up were carried out using AMPure XP® beads, Beckman-Coulter^©^. Then, amplicons were indexed with the Illumina sequencing adapters using the Nextera XT Index Kit® (Illumina^©^) followed by a purification step in the same conditions. Concentration of each indexed amplicon was assessed by Qubit 2.0 Fluorometer (Thermo Scientific^©^), while its quality was evaluated by high-resolution automated electrophoresis (Agilent Bioanalyzer 2100®). Indexed amplicons were sequenced in a paired-end (2 × 300 bp) sequencing format with a MiSeq Reagent Kit V3® (600 cycles), using the MiSeq platform (Illumina^©^).

### Bioinformatics Analysis

Paired end reads 2 × 300 were processed with the QIIME2 pipeline (36). Demultiplexed fastq files were processed with the DADA2 plugin to resolve the amplicon sequence variants (ASVs) (37). Reads were trimmed at position 20 of the 5' end and truncating in position 280 in the 3' end for both forward and reverse reads. Chimeric sequences were removed with the “consensus” method. The taxonomy of each representative sequences of the ASVs was assigned using the QIIME plugin feature-classifier classify-consensus-vs-search (v 2.9.0) (38), using the SILVA database (version 132). The representative sequences of the ASVs were aligned with the MAFFT algorithm (39).

After the masking by positional conservation and gap filtering, a tree was built with the FastTree algorithm (40). The mitochondrial ASVs were removed and the feature table was rarefied at a sequencing depth of 9,800 reads per sample after verifying the correct sample effort by an accumulation curve. The feature table and tree were exported to the R environment v. 3.6.0 (http://www.R-project.org/) and the statistical analyses were performed with the phyloseq v. 3.6.0 (41), ggplot2 v. 3.3.0 (42), and vegan packages v. 2.5.6 (43).

First, beta diversity Permutational Analysis of Variance (PERMANOVA) test with 1,000 permutations with the weighted UniFrac distance was carried out to assess significant differences among treatments. Second, a Principal Coordinate Analysis (PCoA) on weighted UniFrac distance was calculated (44). Third, richness and alpha-diversity were calculated using observed ASVs, Shannon diversity measurements H', Simpson's metric, Chao1's metric, and abundance-based coverage estimator (ACE). Then, a linear discriminant analysis (LDA) effect size (LEfSe) (45) was performed at the ASV level to identify the microbial taxa with differential abundances among treatments, using a LDA cut-off > 2 and a Kruskal-Wallis alpha value *p* < 0.05. Finally, a co-occurrence network analysis was performed by pairwise comparison from the LEfSe results with a correlation analysis using the SparCC software (46) and analyzed using Cytoscape v3.7.2 (47).

### Data Deposition

Raw sequences from 16S rRNA gene profiling are available in the NCBI SRA database with access numbers: SAMN13874135; SAMN13874136; SAMN13874137; SAMN13874138; SAMN13874139; SAMN13874140; SAMN13874141; SAMN13874142; SAMN13874131; SAMN13874132; SAMN13874133 and SAMN13874134; all under the NCBI BioProject PRJNA601771.

### Hydrocarbon Quantification

A sample of the water column was taken for WAF and CEWAF at the beginning (0 h) and at the end (96 h) of the experiment. Total hydrocarbons, aliphatic (C_10_ - C_40_) and PAHs, including 16 US EPA priority PAHs were quantified in WAF and CEWAF following the method of Wang et al. (48). Prior to extraction, samples were enriched with 100 μl of the following standards: biphenyl d10, phenanthrene d10, chrysene d12, benzo(a)pyrene d12 (10 mg/mL), and o-terphenil (200 mg/mL). In each set of samples, a technical blank and a duplicate sample were added. Identification and quantification of the compounds was carried out with standards from Ultra Scientific® in the case of the PAHs and from Chiron^©^ for deuterated PAHs. Total hydrocarbons were analyzed with an Agilent 7890A^©^ gas chromatograph equipped with an FID detector. PAHs were analyzed with a Perkin-Elmer^©^ gas chromatograph equipped with a Clarus 500® mass-selective detector using a 30 m × 0.25 mm (i.d.) x 0.25 DB-5 MS fused silica capillary column (J & W Scientific^©^), operating in the selected ion monitoring (SIM) mode, calibrations were verified daily, and the calibration curves were carried out for each set of samples.

### Statistical Analysis

The total weigh and length of each fish were measured at the beginning and at the end of the experiment (0 and 96 h, respectively), and analyzed by a two-tailed Student's *t*-test with a *p* < 0.05. The metrics of observed ASVs, Shannon and Simpson alpha-diversity indices, Chao1 and ACE richness measurements were tested for significance differences between groups at 96 h exposure. For this, the normality of the data was tested by the Shapiro–Wilk test and a two-tailed Student's *t*-test was performed for parametric data using InfoStat software (49). All data are reported as a mean ± standard error (SEM) and ^*^p < 0.05 and ^**^p < 0.01 were considered statistically significant.

## Results

### General Information and *D. rerio* Biometrics

The acute 96 h exposure to WAF and CEWAF treatments did not alter the total length or weight of fish among groups or between times (*p* > 0.05) (Supplementary Table 1). Water quality parameters were constant along the experiment, including nitrites and ammonium concentrations (${\text{NO}}_{2}^{-}$ = not detected, ${\text{NH}}_{4}^{+}$ = 0.03 ± 0.0 mg/L), temperature (27 ± 0.5°C), dissolved oxygen (DO = 5.50 ± 0.25 mg/L), salinity (S = 0.0 UPS), and hydrogen potential (pH = 5.5 ± 0.0).

### Sequencing Data Analyses

After sequencing the V3-V4 region of the rRNA gene, a total of 1,611,258 reads were obtained from the 12 libraries. After cleaning the raw data, 502,245 reads remained for statistics analysis (Supplementary Table 2A). The percentage of mitochondria detected in the sequencing data was 6%, while the number of chimeric sequences was 3% of the total raw reads.

### Effects of WAF and CEWAF on Richness and Alpha Diversity of Gut-Associated Microbiota of *D. rerio*

**Figure 1:**
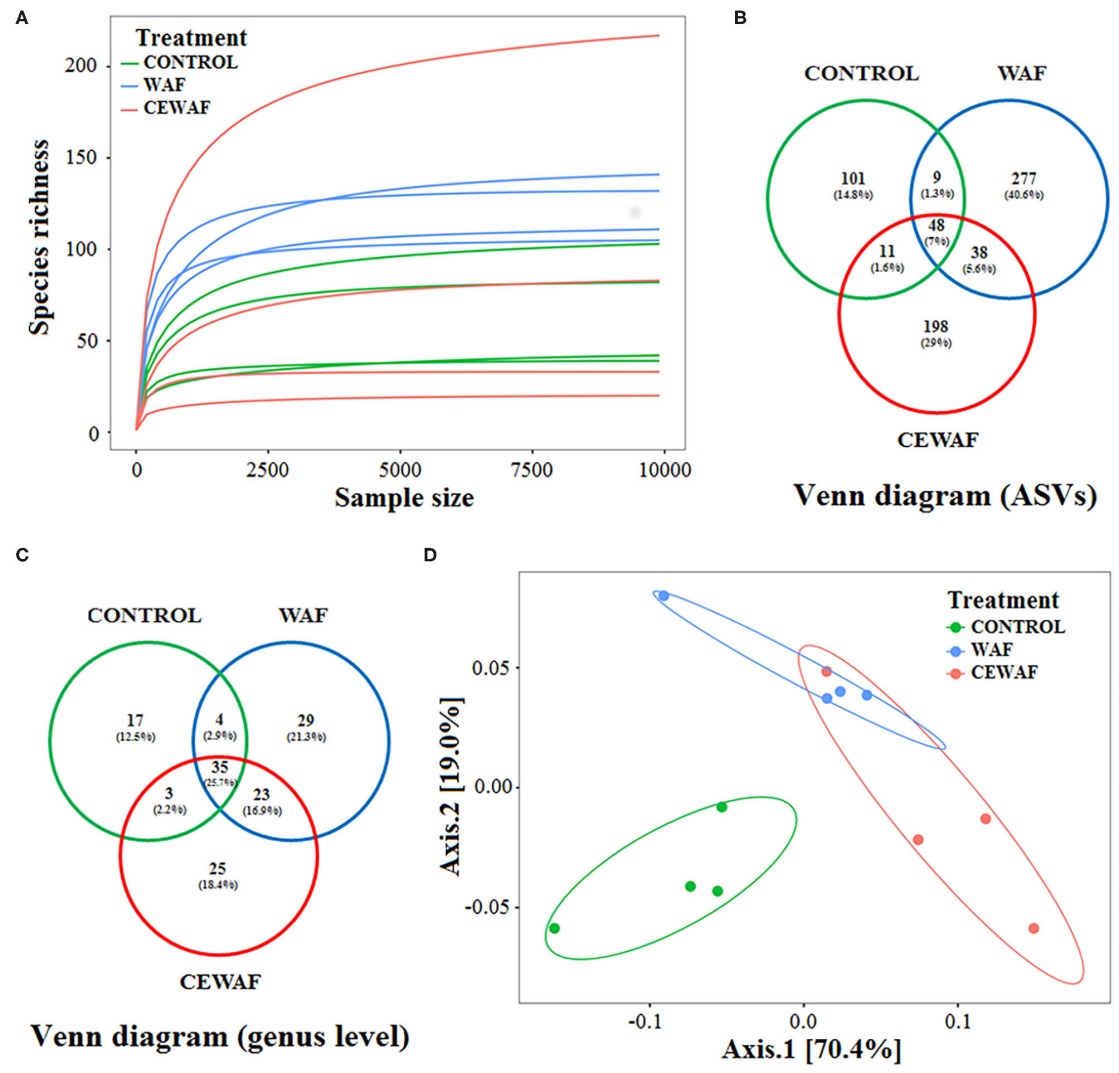
Main statistics of biodiversity analysis. **(A)** Rarefaction curves of ASVs. **(B,C)** Venn diagram of the ASVs that are shared among the groups. **(D)** UniFrac PCoA plot showing the separation among sample groups exposed to WAF (blue dots) and CEWAF (orange dots) respect to CONTROL (green dots) group.

We used rarefaction curves to estimate the correct sampling effort. According to this analysis, all libraries possess a sufficient sequencing depth because all of them reached the plateau phase at 9,800 reads (Figure 1A). Richness and diversity of the gut-associated microbiota of zebrafish were compared among groups. To estimate richness and coverage, Chao1 and ACE indices were used, respectively. Both indices indicated a higher richness for WAF treatment in comparison with CEWAF and CONTROL, with only statistical differences between WAF and CONTROL (*p* < 0.05) (Table 1). In contrast, CEWAF showed a greater variability without significant differences when it was compared with the other groups (Table 1).

**Table 1:** The diversity and richness indexes of the gut microbiota in zebrafish in response to WAF, CEWAF, and control group.

**Group**	**Observed ASVs**	**Diversity**		**Richness**	
		**Shannon**	**Simpson**	**Chao1**	**ACE**
Control	66.50 ± 15.62	2.32 ± 0.20	0.79 ± 0.03	68.13 ± 16.40	68.54 ± 16.12
WAF	122.25 ± 8.52^*^	3.50 ± 0.18^**^	0.93 ± 0.02^*^	123.63 ± 8.75^*^	123.54 ± 8.71^*^
CEWAF	88.25 ± 45.01	2.28 ± 0.64	0.73 ± 0.08	91.33 ± 47.02	91.23 ± 47.20

All data are presented as the mean ± standard error (SEM) of replicates per group,

* p < 0.05 and

** *p < 0.01*.

A total of 682 ASVs were obtained (Figure 1B; Supplementary Table 2B). For CONTROL group, 169 ASVs (24.7%) were registered; for WAF treatment, we identified 372 (54.5%) ASVs, and for CEWAF treatment, a total of 295 (43.2%) ASVs were found (Figure 1B; Supplementary Table 2B), and the classification at genus level is shown in Figure 1C.

### Effects of WAF and CEWAF on the Structure of Gut Microbiota of *D. rerio*

The composition of the gut microbiota of each group was compared using a PCoA analysis based on weighted UniFrac distance among samples (Figure 1D). The two axes of PCoA explained 89.4% of the total variance in bacterial composition, showing a clear separation between ASVs abundances from the gut microbiota of WAF and CEWAF treatments in relation to the CONTROL. However, PCoA did not show a clear separation between WAF and CEWAF. Paired-PERMANOVA showed differences between WAF and CONTROL (*F* = 13.35, *R*^2^ = 0.69, *p* < 0.05), and between CEWAF and CONTROL (*F* = 13.74, *R*^2^ = 0.69, *p* < 0.05). In contrast, no difference was found between WAF and CEWAF (*F* = 3.38, *R*^2^ = 0.36, *p* = 0.11).

### Effects of WAF and CEWAF on the Composition of Gut Microbiota of *D. rerio*

**Figure 2:**
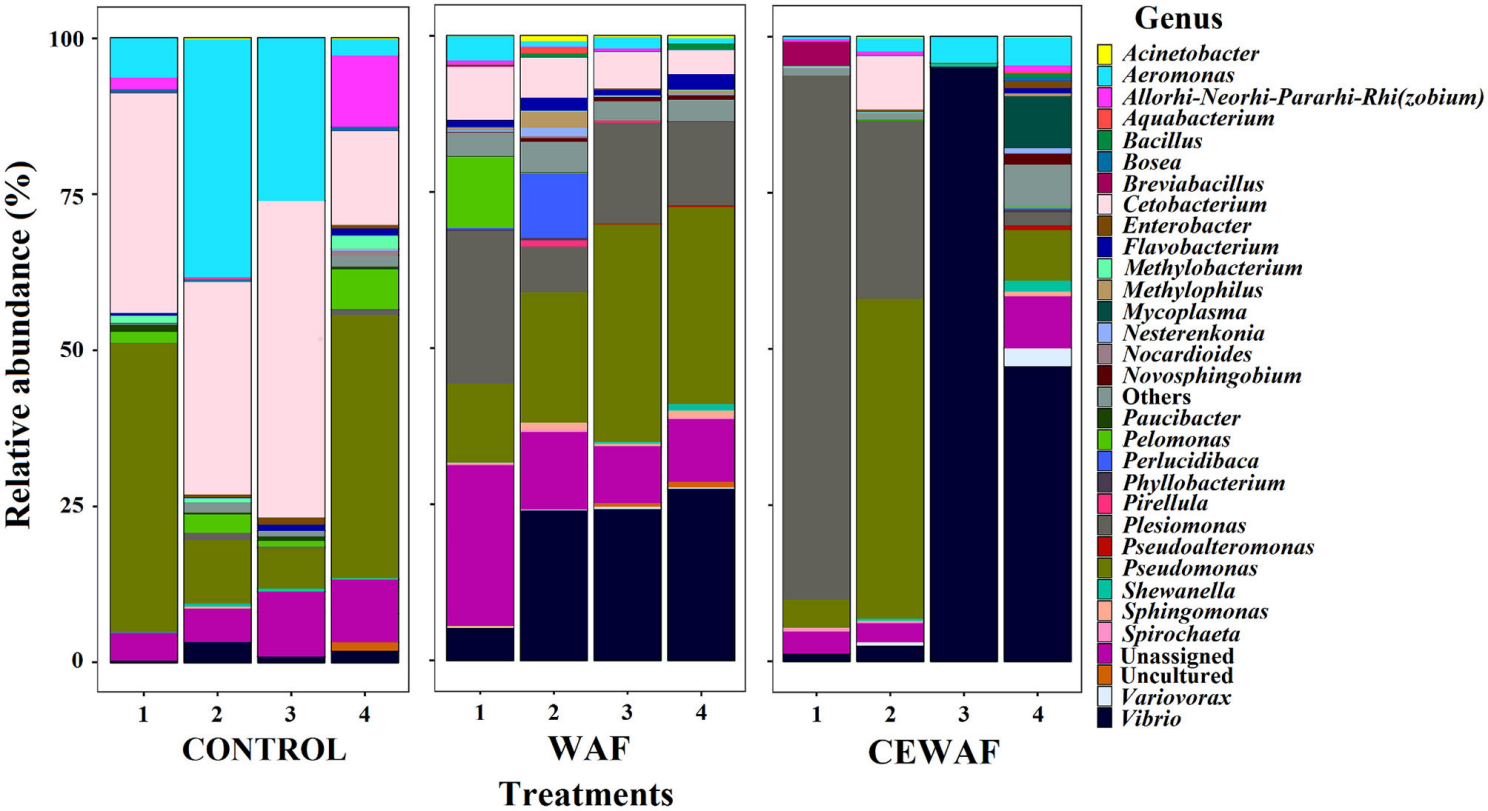
The relative abundance of ASVs classified at genus level (>1%).

A total of 32 bacterial genera with a relative abundance > 1% were identified from all the libraries (Figure 2). The information about the absolute and relative abundances of each genera is described in the Supplementary Tables 2C,D.

Considering the relative abundance, the two major phyla were Proteobacteria (CONTROL: 64.30%, WAF: 83.89%, and CEWAF: 90.90%), and Fusobacteria (CONTROL: 33.73%, WAF: 6.16%, and CEWAF: 2.14%). Chlamydiae (0.04%) was the only phylum specifically found in CONTROL. For WAF and CEWAF, three phyla were found exclusively shared by these treatments: Spirochaetes (WAF: 0.29% and CEWAF: 0.19%), Cyanobacteria (WAF: 0.02% and CEWAF: 0.08%), and Verrucomicrobia (WAF: 0.02% and CEWAF: 0.05%). Finally, Patescibacteria phylum (0.003%) was found only in CEWAF.

The top-three of the genera with highest relative abundance per group were *Cetobacterium* (33.7%), *Pseudomonas* (26.2%) and *Aeromonas* (18.2%) for CONTROL; *Pseudomonas* (24.9%), *Vibrio* (20.2%) and *Plesiomonas* (15.2%) for WAF, and *Vibrio* (36.5%), *Plesiomonas* (28.5%) and *Pseudomonas* (15.9%) for CEWAF (Supplementary Table 2D). The ASVs were distributed among 136 bacterial genera (Figure 1C and Supplementary Table 2B), and the specific genera per treatment are listed in Supplementary Table 2E.

### Differential Abundance Analysis on Microbial Composition of *D. rerio* Gut

**Figure 3:**
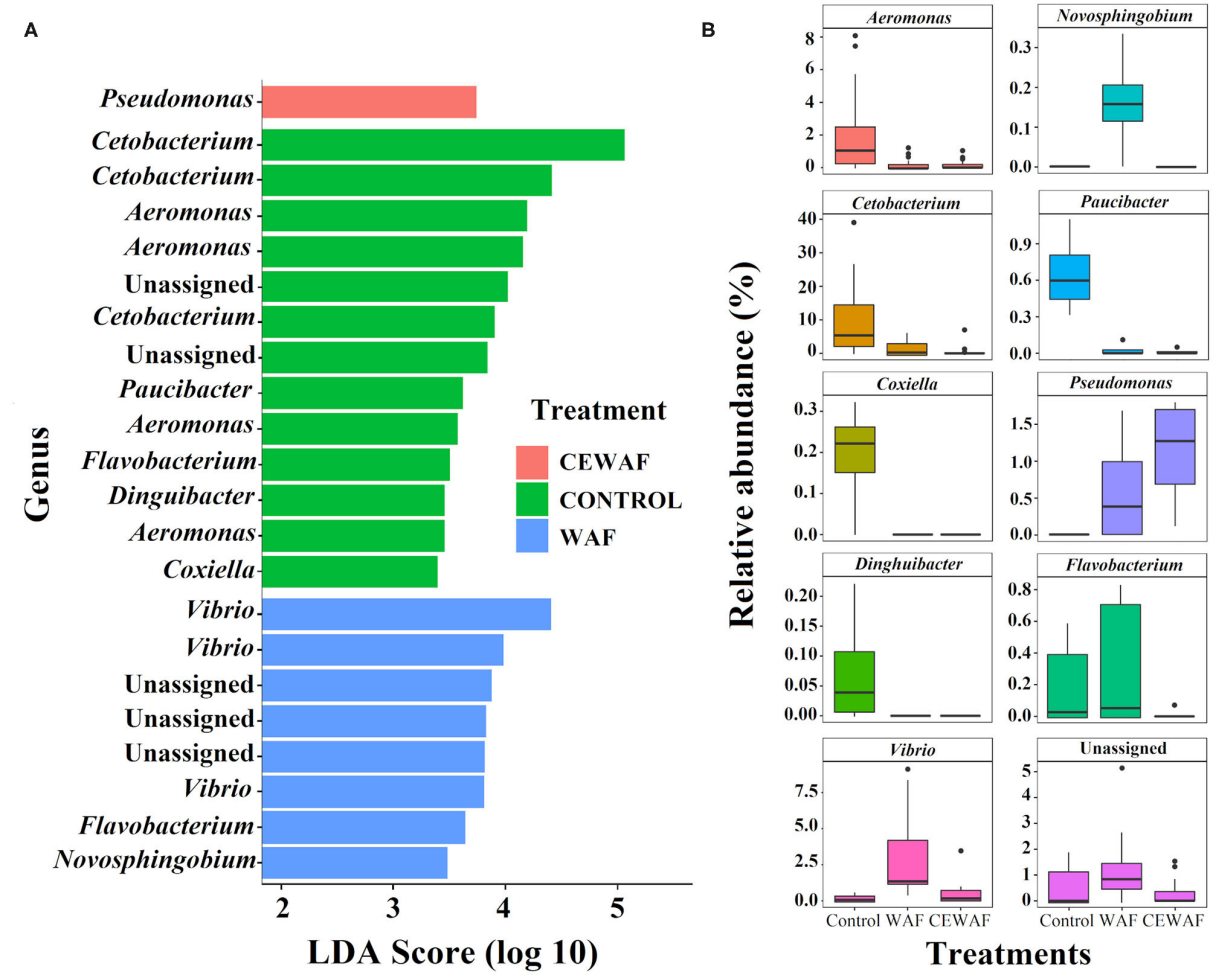
Statistically different phylotypes of the gut-microbiome of *D. rerio* according to LEfSe analysis. **(A)** LDA scores computed for differential ASV's compared among the three groups and represented at genus level. **(B)** The relative abundance of the identified differential ASVs at genus level for each group.

We performed a LEfSe analysis to find the ASVs with differential abundance among the groups (Figure 3A) and by pairwise comparison (Supplementary Figures 1A,C, 2A). Using this approach, 22 representative ASVs were identified among the three conditions, 17 belonging to 9 genera (*Pseudomonas, Cetobacterium, Aeromonas, Paucibacter, Flavobacterium, Dinguibacter, Coxiella, Vibrio*, and *Novosphingobium*, while 5 ASVs were unassigned genera (Figure 3A).

The changes in relative abundances of genera among groups (Figure 3B) and between groups (Supplementary Figures 1B,D, 2B) are shown, and a description of the data analysis are indicated in Supplementary Tables 2F,G,H).

Finally, the phylogenetic analysis performed with the ASVs detected by LefSe analysis between groups identified the closest related species for the ASVs with differential abundance, Supplementary Figure 3 for WAF and Supplementary Figure 4 for CEWAF.

### Microbial Correlation Network Analyses

**Figure 4:**
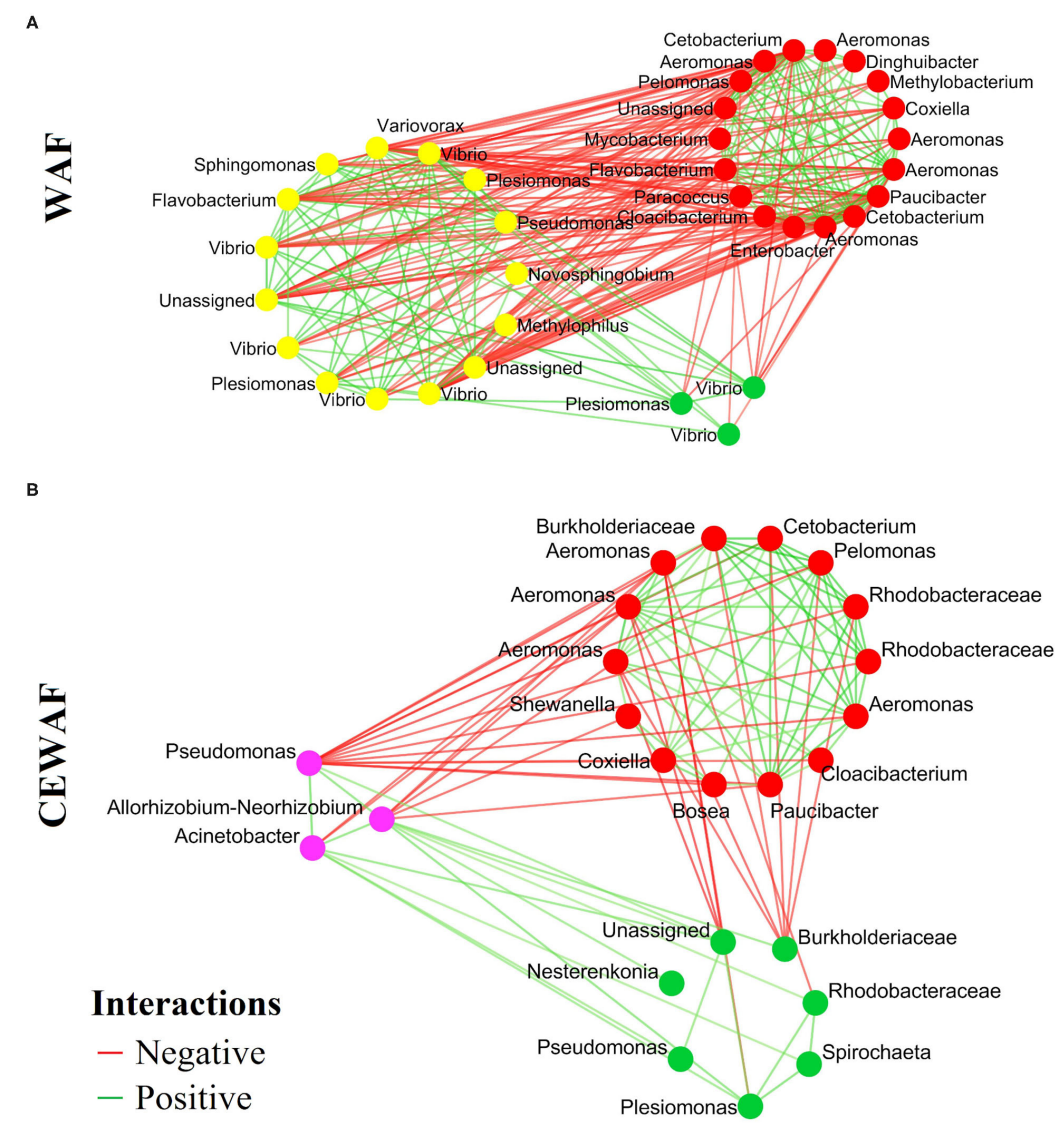
Co-occurrence networks with the ASVs identified in the pairwise LEfSe analysis. Each node means the statistically different genera or family for each condition. **(A)** Representative genera for WAF (yellow dots) and **(B)** CEWAF (purple dots). The red means a negative interactions and the green means a positive interaction among bacterial groups.

The co-occurrence networks based on the differential ASVs show the positive (co-existence) and negative (co-exclusion) interactions of the gut microbiota in response to WAF (Figure 4A) and CEWAF (Figure 4B) treatments. A total of 282 interactions were found (138 negatives and 144 positives) with 36 nodes for WAF (Figure 4A). For CEWAF, we found 100 interactions (30 negatives and 70 positives), with 24 nodes (Figure 4B). The total interactions for the bacterial communities of WAF and CEWAF treatments are shown in the Supplementary Tables 2I,J, respectively.

### Quantification of Hydrocarbons in WAF and CEWAF

The hydrocarbon quantification of WAF and CEWAF was performed at the initial time (0 h) and at the end (96 h) time of the experiment. Four PAHs (naphthalene, 2-methylnaphthalene, 1-methylnaphthalene, and perylene) were quantified for WAF at 0 h with a total concentration of 1.21 μg/L, and for CEWAF, only one PAH (perylene) was detected with a concentration of 0.18 μg/L. In contrast, these PAH were not detected at 96 h. Detailed information on the quantification of each hydrocarbon compound detected at 0 h and 96 h can be found in the Supplementary Table S3.

## Discussion

In this study, we investigated if an exposure to an environmentally relevant concentration of WAF and/or CEWAF could disturb the gut microbiota of *D. rerio* (Supplementary Figure 5). This sort of studies demonstrate that the gut microbiota of fish can be useful in toxicological studies, additional to findings provided in metagenomics analyses from the water-column and sediments (21–23, 50). Previous studies have reported that the basal gut microbiota of *D. rerio* is dominated by the phyla Proteobacteria, and the prevalent presence of Firmicutes and Fusobacteria (11, 51). In this study the CONTROL group harbored the same phyla and were the most abundant. Interestingly, their relative abundance was affected by WAF and CEWAF treatments.

Here, we observed that WAF exposure caused changes in richness, abundance, alpha and beta diversity, dominance, and co-occurrence networks of the gut microbiota. In contrast, CEWAF only impacted the beta diversity, abundance, as well as the interactivity among bacterial taxa. However, both treatments generated dysbiosis. This is because the changes in the alpha diversity appears to be the most consistent indicator of intestinal dysbiosis (52). As well as the increase of the inter-individual variability in the microbiota structure (53–55), that we observed in WAF and CEWAF treated samples, respectively.

We also observed a reduction in the relative abundances of putative beneficial genera. For example, *Cetobacterium* is recognized as a vitamin B12 provider for its host (56). Also, *Lactobacillus* is considered beneficial in the regulation of intestinal functions by secreting metabolites and altering the pH to prevent the settlement of harmful bacteria (57). The reduction of these taxa such as those reported herein, could imply an important loss of resilience in the host microbiota (58, 59). In contrast, *Vibrio, Acinetobacter, Streptococcus, Flavobacterium, Plesiomonas*, and *Pseudomonas* genera are frequently considered pathogenic genera for this model (60–62). Interestingly, their relative abundance increased in both treatments, except for *Flavobacterium* that only increased in WAF treatment. However, *Vibrio* and *Pseudomonas* genera have been reported in the degradation of aromatic compounds (63–65). For example, after the DWH disaster, some members of the *Pseudomonas* genus appeared to be dominant during the oil degradation stage, when the proportions of aliphatic compounds were higher (66). In addition, previous work evaluating surfactants reported an increase in *Pseudonomas* in soils contaminated with hydrocarbons (67).

It is noticeable that although the exposure time was very short (96 h), the 50% WAF treatment was enough to promote an increase in the relative abundance of genera with reported hydrocarbonoclastic capabilities such as, *Acinetobacter, Flavobacterium, Klebsiella, Vibrio, Staphylococcus* and *Shewanella* (68–70). Though, *Achromobacter* was found only increased in response to WAF treatment, this genus has been found in the degradation of hydrocarbons such as n-alkanes and PAHs (71), while Bacosa et al. (72) described that it uses some metabolites from aromatic hydrocarbons.

Another bacterium that increased its abundance in both treatments was *Bacillus*, with some members capable to metabolize hydrocarbons. *Bacillus* is commonly found in crude oil-affected marine areas (73, 74). Likewise, *Acinetobacter* increased in both treatments, and several members of this genus has been proposed as a key player in PAHs degradation processes (75). Also, *Sphingomonas* were reported to have PAHs degradation abilities (76). *Burkholderia* was found only in WAF and CEWAF. This genus has been reported of having a grading capacity to degrade heavy crude oil (77). Thus, the increase of its abundance in response to light crude oil warrants further investigations because the genus *Burkholderia* has over 90 species reported, and it is divided into two major groups phylogenetically distant. The first group is composed of pathogenic species which highlights referred opportunistic pathogens such as *Burkholderia cepacian* (Bcc) complex, and the other group consists of non-pathogenic species with skills to promote plant growth and rhizoremediation (77).

On the other hand, *Muricauda* was found exclusively in organisms exposed to CEWAF. It has been reported as a degrader of aliphatic hydrocarbons (78), as well as *Cycloclasticus* and *Oleiphilus*, were found only in response to CEWAF. These genera have been reported as oil degraders and are frequently found in polluted marine environments (79, 80).

The co-occurrence patterns provide a new perspective to understand the structure of complex microbial communities (26, 81, 82). The bacterial interactions of the differential genera reported herein indicate that both treatments (WAF and CEWAF) can alter in different way the interactions among bacteria. This analysis suggests a greater interactivity in the gut microbiota of *D. rerio* exposed to WAF with respect to CEWAF. This feature in associated with a greater number of bacterial groups, specifically those with putative hydrocarbonoclastic activity, such as *Flavobacterium, Pseudomonas, Novosphingobium, Sphingomonas, Vibrio, Methylophilus, Plesiomonas*, and *Variovorax* (83–85). According to our data, it is likely that as a mechanism of defense, the gut microbiota rearranges their capabilities to enhance its hydrocarbon assimilation capabilities.

In this way, representative groups, helped to increase the presence of other groups of bacteria, triggering a positive synergy with organisms with similar function (like the intake of hydrocarbons as energy source). The alterations observed herein suggest the establishment of an ecological succession of the microbiota that use crude-oil derived compounds, as described previously (80), and have an active role for the metabolism of hydrocarbons in the gut microbiota, during the crude-oil exposure (24). Despite this, it is likely that during this process of biotransformation of hydrocarbons in the gut, the resulting metabolites also increase harmful bacteria that would negatively affect fish health (86).

Our results also indicated that the addition of Nokomis 3-F4® was able to induce a differential assembly as well as bacterial interactions in fish gut microbiota with respect to exposure to WAF. In this sense, chemical surfactants not only affect the distribution of crude oil-derived compounds in the water column, but also can trigger a differential response at the gut microbiota level. Results from this study are encouraging and future studies should focus on the evaluation of the gut metabolic process that take place in response to WAF and CEWAF at different times of exposure and concentrations.

## Conclusion

This is the first study evaluating the effects of light crude oil (WAF), and its mixture with Nokomis 3-F4® (CEWAF) on the gut microbiota of zebrafish, *D. rerio*. We observed that both treatments caused dysbiosis. We found changes in the diversity and abundances of gut microbiota for WAF and CEWAF treated groups. Moreover, these treatments triggered an increase in the abundance of hydrocarbonoclastic genera. These findings have environmental relevance regarding the assessment of the impact of acute exposure to water soluble compounds of crude oil and its mixture with chemical dispersant.

## Data Availability Statement

The datasets generated for this study were uploaded to the Sequence Read Archive (SRA) of the National Center for Biotechnology Information (NCBI) database with access number of the BioProject PRJNA601771 with a release date at 2020-08-03.

## Ethics Statement

The animal study was reviewed and approved by The animal study was reviewed and approved by the Institutional Animal Care and Use Committee of the Center for Research and Advanced Studies (Centro de Investigación y de Estudios Avanzados del IPN) (CICUAL-Cinvestav) (APPROVAL NUMBER: 2875). And comply with the applicable Mexican Official Norm (NOM-062-ZOO-1999), Technical Specifications for the Care and Use of Laboratory Animals, as well as all applicable federal and institutional regulations.

## Author Contributions

CG-P, JZ-B, and RR-C: conceptualization and experimental design. CG-P, JZ-B, and DC-G: metagenomic analysis and data curation. CG-P, JZ-B, RR-C, MA-P, DC-G, and EH-N: writing and editing. CG-P and JP-V: sampling laboratory coordination. RR-C: project administration and funding acquisition. CG-P and JZ-B: formal analysis. CG-P, JZ-B, JP-V, and MA-P: methodology. RR-C and EH-N: supervision. CG-P, JZ-B, DC-G, and MA-P: visualization. All authors contributed to the article and approved the submitted version.

## Conflict of Interest

The authors declare that the research was conducted in the absence of any commercial or financial relationships that could be construed as a potential conflict of interest.

## Supplementary Material

Supplementary Figure 1 LEfSe analysis of the ASV shown significant differential genera between conditions. **(A)** ASV identified at genus level with differential abundance for WAF treatment. **(B)** Differential abundances between WAF and CONTROL. **(C)** ASV at genus level with differential abundance for CEWAF treatment. **(D)** Differential abundances between CEWAF and CONTROL.

Supplementary Figure 2 LEfSe analysis of the ASV shown differential genera between WAF and CEWAF. **(A,B)** ASVs at genus level with differential abundance.

Supplementary Figure 3 Phylogenetic tree of differential, unassigned genera of WAF (yellow) and CONTROL (blue) highlighting their closest relative species.

Supplementary Figure 4 Phylogenetic tree of differential, unassigned genera of CEWAF (purple) and CONTROL (blue) highlighting their closest relative species.

Supplementary Figure 5 Conceptual map referring to gut microbiota response of zebrafish exposed to WAF and CEWAF.
