# Supplementary figures and images for: Alterations in the Gut Microbiota of Zebrafish (Danio rerio) in Response to Water-Soluble Crude Oil Components and Its Mixture With a Chemical Dispersant
Source: Front Public Health. 2020 Oct 26;8:584953. doi: 10.3389/fpubh.2020.584953 (PMC7649143; doi:10.3389/fpubh.2020.584953)

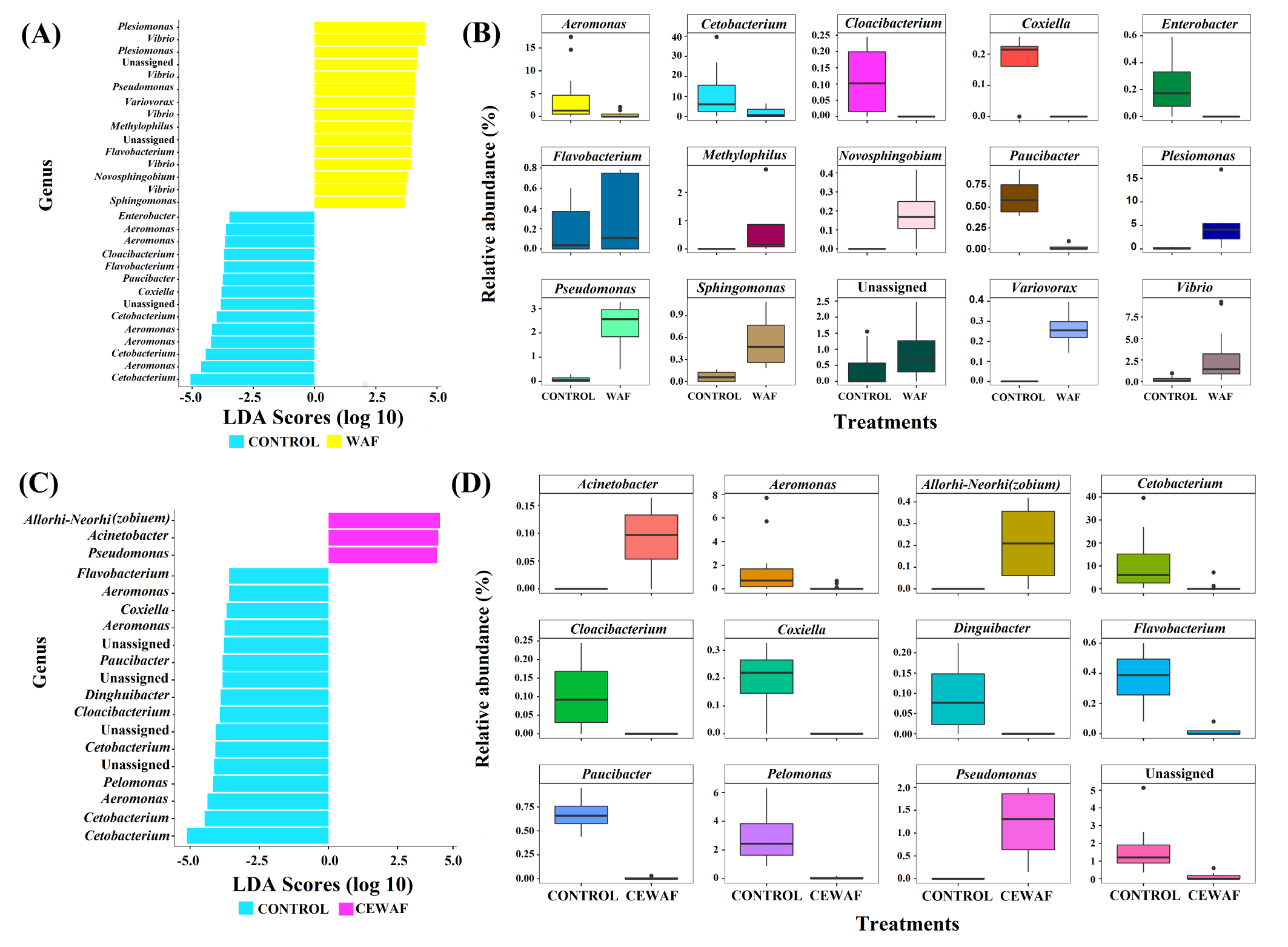

Supplement: Supplementary Figure 1 — LEfSe analysis of the ASV shown significant differential genera between conditions. (A) ASV identified at genus level with differential abundance for WAF treatment. (B) Differential abundances between WAF and CONTROL. (C) ASV at genus level with differential abundance for CEWAF treatment. (D) Differential abundances between CEWAF and CONTROL. [file Image_1.TIF]

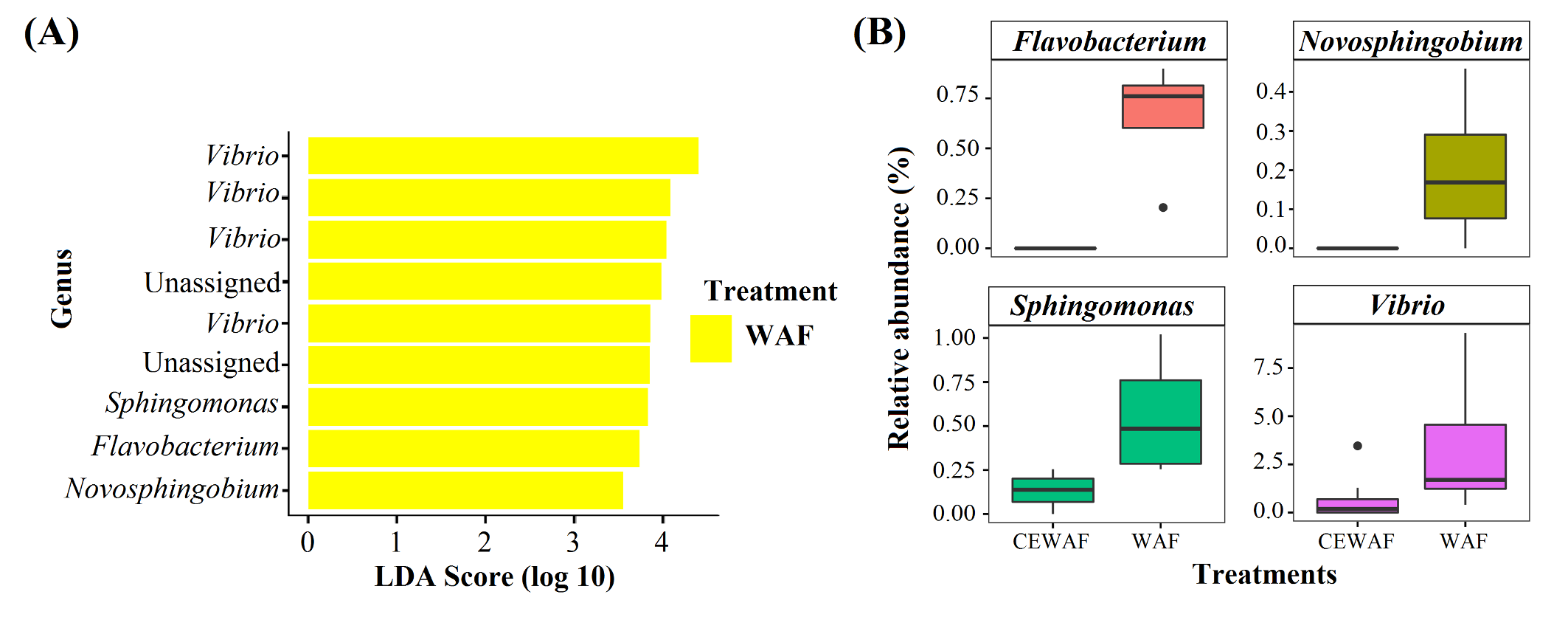

Supplement: Supplementary Figure 2 — LEfSe analysis of the ASV shown differential genera between WAF and CEWAF. (A,B) ASVs at genus level with differential abundance. [file Image_2.TIF]

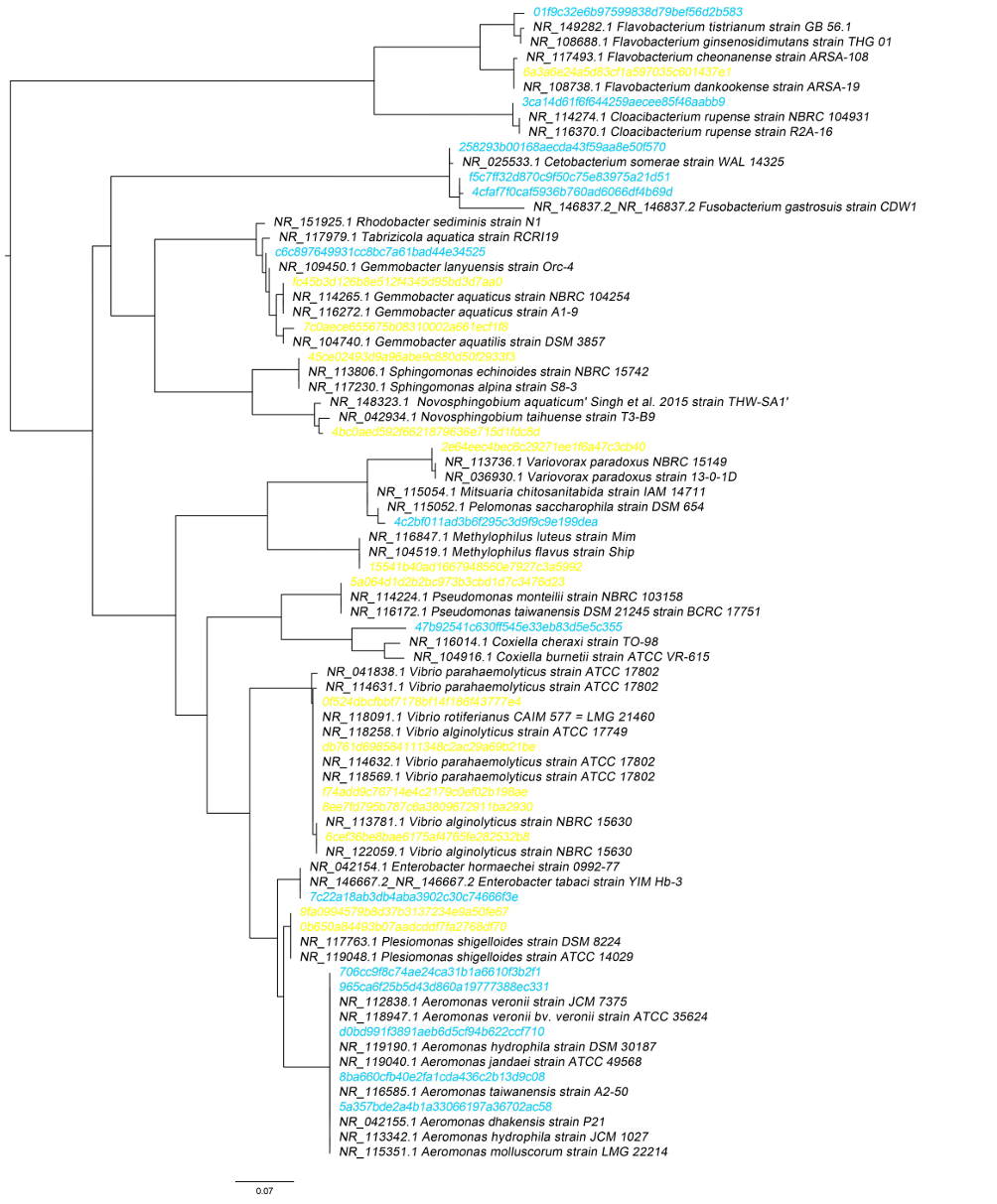

Supplement: Supplementary Figure 3 — Phylogenetic tree of differential, unassigned genera of WAF (yellow) and CONTROL (blue) highlighting their closest relative species. [file Image_3.TIF]

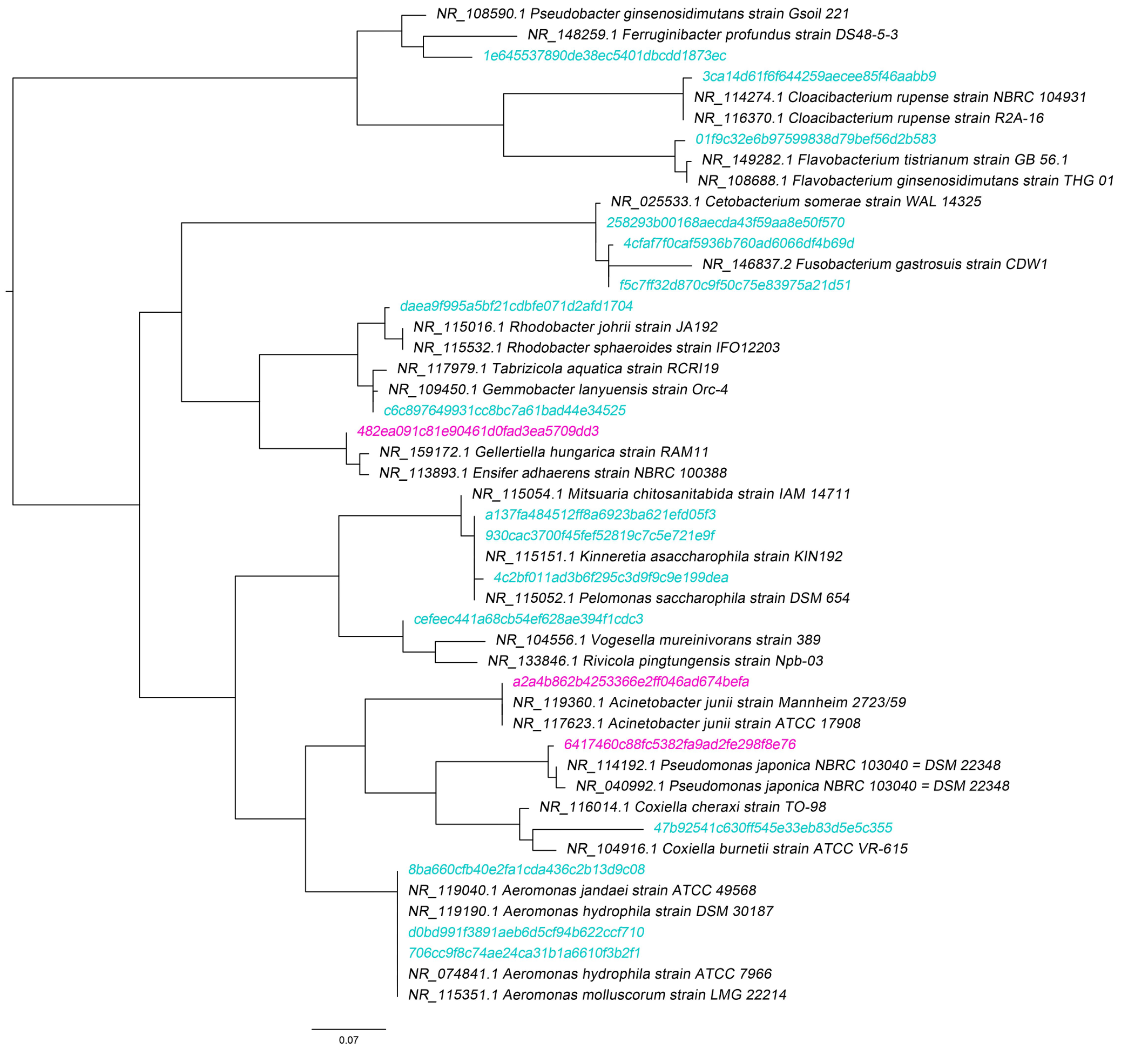

Supplement: Supplementary Figure 4 — Phylogenetic tree of differential, unassigned genera of CEWAF (purple) and CONTROL (blue) highlighting their closest relative species. [file Image_4.TIF]

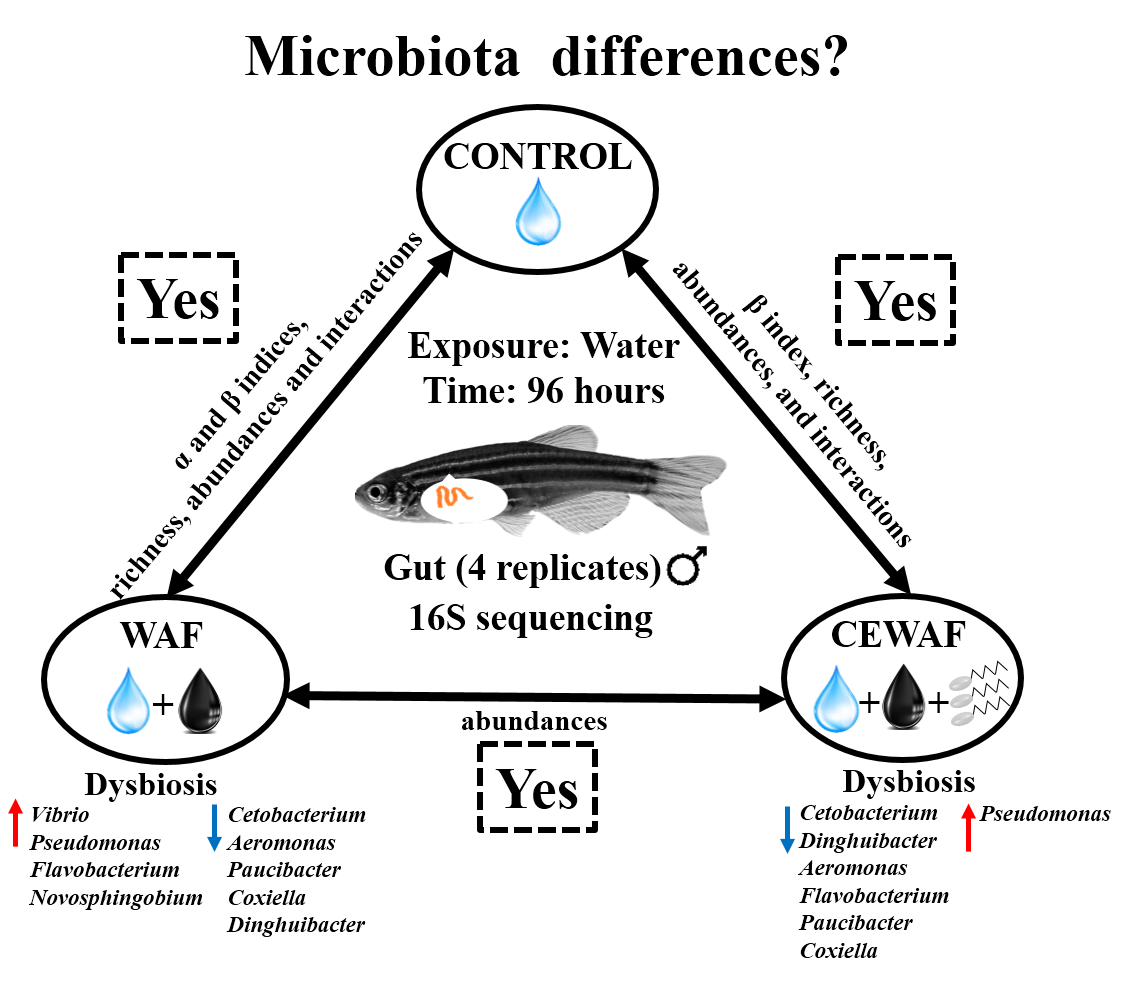

Supplement: Supplementary Figure 5 — Conceptual map referring to gut microbiota response of zebrafish exposed to WAF and CEWAF. [file Image_5.TIF]
